# Functional Connectivity Estimated from Intracranial EEG Predicts Surgical Outcome in Intractable Temporal Lobe Epilepsy

**DOI:** 10.1371/journal.pone.0077916

**Published:** 2013-10-30

**Authors:** Arun R. Antony, Andreas V. Alexopoulos, Jorge A. González-Martínez, John C. Mosher, Lara Jehi, Richard C. Burgess, Norman K. So, Roberto F. Galán

**Affiliations:** 1 Epilepsy Center, Cleveland Clinic, Cleveland, Ohio, United States of America; 2 Department of Neurosciences, School of Medicine, Case Western Reserve University, Cleveland, Ohio, United States of America; University of Michigan, United States of America

## Abstract

This project aimed to determine if a correlation-based measure of functional connectivity can identify epileptogenic zones from intracranial EEG signals, as well as to investigate the prognostic significance of such a measure on seizure outcome following temporal lobe lobectomy. To this end, we retrospectively analyzed 23 adult patients with intractable temporal lobe epilepsy (TLE) who underwent an invasive stereo-EEG (SEEG) evaluation between January 2009 year and January 2012. A follow-up of at least one year was required. The primary outcome measure was complete seizure-freedom at last follow-up. Functional connectivity between two areas in the temporal lobe that were sampled by two SEEG electrode contacts was defined as Pearson’s correlation coefficient of interictal activity between those areas. SEEG signals were filtered between 5 and 50 Hz prior to computing this correlation. The mean and standard deviation of the off diagonal elements in the connectivity matrix were also calculated. Analysis of the mean and standard deviation of the functional connections for each patient reveals that 90% of the patients who had weak and homogenous connections were seizure free one year after temporal lobectomy, whereas 85% of the patients who had stronger and more heterogeneous connections within the temporal lobe had recurrence of seizures. This suggests that temporal lobectomy is ineffective in preventing seizure recurrence for patients in whom the temporal lobe is characterized by weakly connected, homogenous networks. This pilot study shows promising potential of a simple measure of functional brain connectivity to identify epileptogenicity and predict the outcome of epilepsy surgery.

## Introduction

Approximately one third of patients with epilepsy are intractable to medical therapy and surgery becomes an alternative option. Resective and non-resective surgical strategies have been used to attain seizure freedom in a proportion of these patients. Despite the advances in diagnostic imaging and electrophysiological techniques, the surgical outcome remains modest at best. Only 47–53% patients with temporal lobe epilepsy (TLE) remain seizure free, 10 years after anterior temporal lobectomy[1]–[3]. Consequently, the development of better tools to localize the epileptogenic zone and guide resection with greater precision is of paramount importance.

The epileptogenic zone is a theoretical concept and is defined as the area of cortex that is indispensable for the generation of epileptic seizures, which on total removal or disconnection renders the patient seizure free [4]. While this idea is still valid and practical, the concept of epilepsy as a localized region of abnormality has since evolved to a disease of cortical networks with nodes and connections involving even regions farther from the seizure onset zone [5]–[7]. Altered perturbations and interactions between various nodes in multiple cortical networks in epilepsy have been intensively studied in the past few years. In order to detect and quantify these interactions, several measures of functional connectivity have been proposed. By “functional”, it is implied that connectivity is operationally defined, according to the tools used to quantify statistical interdependences between simultaneous recordings from different brain areas. A plethora of quantitative methods have been used to analyze functional connectivity with signals from EEG, MEG and fMRI [8], [9], and in particular, in the context of epileptogenesis, seizure propagation and classification of epilepsy [10]. Altered connectivity in different brain regions has been reported in childhood absence epilepsy, juvenile myoclonic epilepsy, photosensitive epilepsy syndromes and focal epilepsies like TLE [11]–[14].

In this context, previous studies have shown that measures of synchrony in interictal intracranial EEG remain stable over long periods of time and can be used to detect seizure onset zone [15]. Methods like cross-correlation and coherence were applied to EEG signals decades ago to study seizure spread and inter-hemispheric interactions in focal epilepsy [16]–[19]. Surgical removal of sharply defined synchronization clusters in electrocorticography from lateral temporal cortex of patients with intractable epilepsy assessed by linear correlation, phase synchronization and mutual information correlated with seizure outcome [20]. In a small sample of nine patients with medically intractable epilepsy studied using subdural grids, Schevon and collaborators found that resection of regions of local hypersynchrony between adjacent pairs electrodes measured by mean phase coherence improved seizure outcome [21]. Similar findings were noted in a recent study by Warren and colleagues who showed that linear cross-correlation and mean phase coherence were high inside the seizure onset zone. Importantly, they noted lower synchrony between seizure generating areas and the surrounding brain regions using the same measures in the local field potential recorded from intracranial EEG [22]. They concluded that in focal epilepsy, the seizure onset zone is functionally isolated from the surrounding brain regions.

In recent computational studies, virtual brains modeling diseased states like epilepsy displayed lower structural complexity compared to models of normal neural function [23]. A measure of neuronal complexity loss estimated from subdural electrodes during presurgical evaluation of epilepsy correctly identified 86.7% of the patients with respect to seizure outcome [24], consistently with other reports [25].

Along the lines of these previous studies on correlated neural activity and network complexity, we investigated the utility of normalized cross-correlations (Pearson’s correlation coefficient) between SEEG signals as a measure of functional brain connectivity to determine if the strength and heterogeneity of functional connections predict surgical outcome of temporal lobectomy in intractable temporal lobe epilepsy. Cross-correlations provide an intuitive and computationally efficient measure of the global entrainment between two signals, which makes them a natural choice for a pilot study.

To our knowledge, no other studies have attempted to shape a surgical outcome measure based on both strength and variability of functional connectivity. We hypothesized that assessing functional connectivity of both mesial and neocortical temporal regions together using SEEG would enhance predictability of our measure.

## Methods

### Ethics Statement

Our research protocol was approved by the Institutional Review Board at the Cleveland Clinic (protocol number and title: IRB 12–186; Evaluation of Functional Connectivity Using EEG Signals in Patients with Intractable Epilepsy). All data were processed anonymously. Written consent by the patients was waived by the IRB for this retrospective study.

### Patient Selection and Exclusion Criteria

Clinical and electrophysiological data of all patients who underwent an intracranial EEG evaluation using SEEG electrodes between January 2009 and January 2012 were reviewed (n = 149). Patients who had intractable TLE and underwent a standard anterior temporal lobectomy with one year follow-up were included in this study (n = 23). All patients underwent appropriate pre-surgical evaluation with noninvasive video electroencephalography evaluation, high resolution brain MRI (Siemens 1.5 Tesla SP system, Erlangen, Germany) using a standardized epilepsy protocol, PET scan and detailed neuropsychology evaluation. The recommendation to proceed to an invasive evaluation was made during a multidisciplinary patient management conference, typically for localization of the ictal onset zone in patients with conflicting non-invasive data or to rule out pseudo-TLE. Multiple electrodes were placed using a robotic system (ROSA™, Medtech, Montpellier, France) according to a pre-implantation hypothesis about the possible epileptogenic zone. Each electrode had 10 cylindrical platinum contacts which were 2–3 mm long and 0.89 in diameter (Ad-tech, Racine, WI, USA). A favorable outcome was defined as complete seizure-freedom one year after surgery. Patients who had topectomies, temporal pole resection or incomplete resection the anterior temporal lobe were not included to facilitate the comparison between patients.

### Data Acquisition and Preprocessing

**Figure 1:**
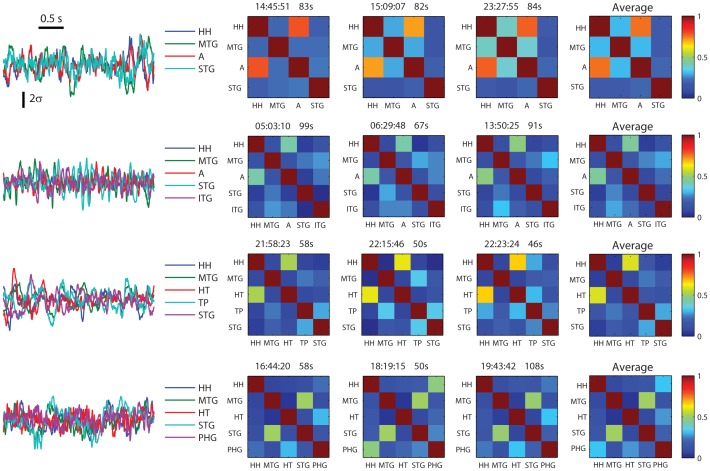
Connectivity matrices are consistent over time. Left: sample traces of SEEG for four patients. Amplitude is in standard deviation units (z-score). Right: Connectivity matrices for four patients (top to bottom) and their small changes over time (left to right).

To identify the areas where electrode contacts were placed, the temporal lobe was first parcellated into 9 standardized regions, namely: hippocampus head (HH), hippocampus tail (HT), amygdala (A), superior temporal gyrus (STG), medial temporal gyrus (MTG), inferior temporal gyrus (ITG), fusiform gyrus (FG), parahippocampal gyrus (PHG), and temporal pole (TP). Recordings were not obtained from all the 9 parcellated regions in the same patient due to lack of electrode contacts as the implantation schema differed slightly between patients depending on the results of the non-invasive evaluation. The location of the contacts was then compared visually to publicly available parcellation maps, allowing us to identify the areas from which the recordings were made in the temporal lobe [26], [27]. Parcellation of the temporal lobe was not automated. Figure S1 shows the corresponding parcellated regions. SEEG traces were recorded at sampling rates of 1, 0.5, or 0.2 kHz depending on the recording and storage capabilities available when the patient was evaluated. The SEEG traces were off-line filtered with a custom-made digital filter in the frequency domain (pass band: 5 to 50 Hz; stop frequencies: 0 and 60 Hz). From the recordings of each patient, we selected three non-overlapping segments of interictal activity of approximately 90 s; sometimes a bit shorter or longer so that intervals with repetitive interictal spikes were avoided in each segment. The segments were taken at different times over a time span of one to several hours (examples shown in Fig. 1). We selected samples prior to the first seizure to avoid sampling segments with high synchrony due to the ictus. Samples within 30 minutes of the seizure were not selected for analysis to avoid pre-ictal changes in EEG [28]. The state of the patient (awake, drowsy, sleep) and medication withdrawal were not taken into account during selection of SEEG segment for analysis. Our analysis was restricted to SEEG signals from the temporal lobe for consistency across patients, even though the anatomical locations of electrode placement also included the orbitofrontal region, insula, cingulate or precuneus. Spike-free (or almost free) segments were selected and any residual spikes were “clipped”. The difficulty in choosing an ideal reference is well known and various methods have been suggested to overcome it [29], [30]. We used the ‘0 V’ reference (effective ground) provided by our SEEG recording system (Nihon Kohden, Tokyo, Japan) which is a linked C3/C4 reference. Using simulation studies, Rappelsberger found that the most reliable results for evaluation of coherence were obtained when this referential montage was used compared to bipolar, common average or source derivation recording [31].

### Data Analysis and Functional Connectivity

Functional connectivity between two areas in which two electrode contacts were located was defined as the normalized covariance of their recorded traces. More generally, and quantitatively, consider a set of *n* simultaneous recordings of intracranial EEG activity, denoted by 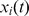 where the subindex refers to the *i*-th trace 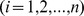, and *t* indicates the ordinal time stamp of the recordings 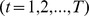. The functional connectivity matrix between area *i* and area *j* is defined as Pearson’s correlation coefficient between 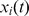 and 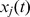.
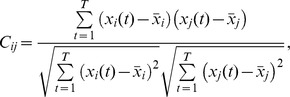
where the mean value of 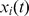 is given by 
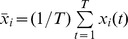
. Note that the values of all matrix elements are bounded between −1 and 1. Also, all diagonal elements are 1 by definition (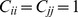) and are irrelevant for our analysis.

### Support Vector Classifier

**Figure 2:**
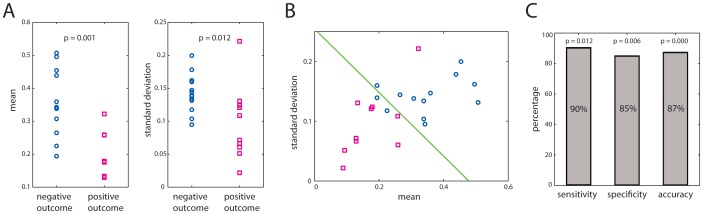
Predicting surgical outcome from connectivity. **A)** The mean connection strength and the variability of the connections are both significantly lower in patients with a positive outcome. B) When used in combination, these two parameters can resolve both groups of patients with only 13% error. C) The sensitivity, specificity and accuracy of the patient classification are highly significant.

For each patient, we calculated the mean and standard deviation of the off-diagonal elements in the connectivity matrix. These two values respectively correspond to the *x* and *y* coordinates of each point in Fig. 2B. A support-vector classifier (SVC) is a standard algorithm to optimally separate two labeled datasets from each other [32]. In the case of two dimensions, as in Fig. 2B, this translates into finding a line that separates the plane in two halves. If a perfect separation is possible, each half will contain data points of one label but not the other. When the separating line is constrained to be straight, as is the case in our study, the SVC is equivalent to a linear discriminant analysis. The two labels (categories) used in our analysis are whether the patient had a positive (seizure freedom) or negative (seizure recurrence) outcome after temporal lobectomy.

### Accuracy, Sensitivity and Specificity of the Classifier

SVCs have the advantage over clustering methods of tolerating some overlap (soft margin) between datasets. When overlap exists, the mere existence of a separating line is not very informative, since both groups are not 100% separable. In such cases, the classification performance index, also known as accuracy (see next section), is a better parameter to quantify the goodness of the discrimination, which is computed with the leave-one-out method. First, the separating line is computed after removing one point from the data set. Then, one tests if the point that was left out is correctly classified. These three steps (removal of a point, calculation of the discriminator and classification of the point removed according to that discriminator) are then iterated for all data points. The fraction of points that are correctly classified for each stimulus is the accuracy of the classifier. High accuracy means that the separating manifold is fairly insensitive to the removal of any given point, and hence robust to perturbations of the data set. This in turn implies that the space in which the points are represented is divided in two category-specific halves, despite some overlap between datasets.

Using the leave-one-method, one can also compute the sensitivity and specificity of the SVC, which are computed from the number of true and false positives (TP and FP, respectively) and true and false negatives (TN and FN, respectively), which in our case are defined as: TP, surgical outcome for the patient that was left out prior to the calculation of the separating line is positive (seizure freedom) and the SVC predicts a positive outcome for that patient; FP, surgical outcome is negative (seizure recurrence) and the SVC predicts a positive outcome; TN, surgical outcome is negative and the SVC predicts a negative outcome; FN, surgical outcome is positive and the SVC predicts a negative outcome. One then has: 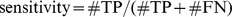,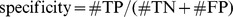, 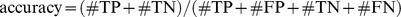, where “#” means “number of”.

### Random Permutation Test (Cross-validation of the SVC)

To determine whether the sensitivity, specificity and accuracy values of the SVC are statistically significant, we use a shuffling approach by randomizing the outcome of surgery, which is a binary variable (1: successful surgery; 0: unsuccessful surgery). Specifically, we take a random permutation of the 0’s and 1’s attributed to the 23 patients. For each randomization we recomputed the SVC, its sensitivity, specificity and accuracy using the leave-one-out method. We repeat the process for 10,000 randomizations and compute the probability (p-value) of obtaining a sensitivity, specificity and accuracy values equal to or higher than those obtained from the actual data. These p-values are reported in Fig. 2C.

## Results

The clinical characteristics of all the 23 patients who underwent standard temporal lobectomy and were considered in our study are summarized in Table S1. Briefly, the patients’ age ranged from 18 to 59 years and 48% were male. 65% of the patients had a normal MRI. Three patients had prior epilepsy surgery and 9 patients had multiple seizure types. Nine patients were found to have focal cortical dysplasia on pathology and five patients had hippocampal sclerosis in addition to focal cortical dysplasia. Only 43.48% remained seizure free at the end of one year of follow up. The high rate of seizure recurrence reflects the expected low rates of seizure-freedom in patients whose non-lesional, poorly localized, intractable epilepsy necessitates evaluation using invasive electrodes.

Figure 1 shows representative four-second long traces of interictal activity from SEEG recordings (left) for four different patients (top to bottom). Traces in different colors represent records from different areas in the temporal lobe, as indicated. Even though these samples were arbitrarily chosen, the fluctuating activity clearly shows some level of entrainment between certain pairs within the same patient. This entrainment is quantified in the matrices plotted next to the right. The matrices display the pair-wise correlation coefficients computed over three non-overlapping segments of the recordings starting at different times (indicated at the top, left in hours: minutes: seconds) with a duration of ca. 90 s (indicated at the top, right in seconds). The fourth matrix on each row is the average of the correlation matrices for the three segments in that patient. Overall the correlation matrices are fairly stable over time as they are consistent across different segments.

A detailed inspection of the averaged connectivity matrices for all patients reveals that, with few exemptions, patients who had a positive outcome after surgery had weaker connections and more homogeneous in strength (see Figure S2). To quantify this observation, we computed the mean and standard deviation of the off-diagonal elements of the averaged connectivity matrices for each patient. Figure 2A (left) shows the mean connectivity values for all patients with negative (left) and positive (outcome). Patients with a positive outcome tend to have smaller values of the mean connection strength. The difference of the medians is statistically significant (p = 0.001; Wilcoxon sum-rank test). Similarly, Fig. 2A (right) shows the standard deviation of the connectivity values for all patients. The variability of the connection strength also tends to be smaller in patients with a positive outcome. Despite one outlier, the difference of the medians is also significant (p = 0.012; Wilcoxon sum-rank test). When the mean connectivity and its standard deviation are considered at the same time, the separation between both groups of patients becomes even more apparent, as shown in Fig. 2B. A linear discriminator can separate the data points from each group with 3 misclassified patients out of 23 (13% error). We found that 90% of the patients who had weak and homogenous connections were seizure free, whereas 85% of the patients who had stronger and more heterogeneous connections within the temporal lobe had recurrence of seizures. Using a cross-validation test based on the leave-one-out method and random permutations (see Methods), we computed the sensitivity, specificity and accuracy of the linear discriminator, which yields the results shown in Fig. 2C. We note that none of the 20 parameters shown in the Table S1 summarizing the patients’ clinical data is significantly different between both groups (all p-values are larger than 0.05). This implies that functional connectivity, as defined here, adds an important prognostic value to currently used parameters in clinical practice.

Finally, we investigated if the functional connectivity between specific pairs averaged across patients correlated with the surgical outcome. Figure S3 shows the averaged connectivity values between areas for the positive (left) and negative (right) outcomes. It is again evident that a positive outcome is overall associated with low connectivity values except for the interaction between the hippocampus and amygdala which is on average above 0.5. High connectivity values are in general associated with a negative outcome, especially between the parahippocampal gyrus and the temporal pole. Entries shaded in black in the connectivity matrices correspond to pairs that were not present in our pilot study. A larger dataset would be necessary to evaluate if the functional connectivity between specific pairs also has a predictive value for the outcome of temporal lobe epilepsy.

## Discussion

Our study shows that the matrices of functional connectivity have a predictive power for the outcome of epileptic surgery. Our study showed that 90% of the patients who had weak and homogenous connections were seizure free, whereas 85% of the patients who had stronger and more heterogeneous connections within the temporal lobe had recurrence of seizures In particular, by looking at the mean and standard deviation of the functional connections it is possible to predict the outcome with 87% accuracy. This is quite remarkable considering that our analysis is based on two assumptions: 1) there is only one epileptogenic zone in the brain that is located in the temporal lobe; 2) the electrodes properly sampled that zone or its immediate neighbors. The main reason for the prediction error is probably due to the existence of more than one epileptogenic zone in our patient population, which explains the high seizure recurrence rate. Even so, the specificity and accuracy of our predictions are well above the 99% confidence level and the sensitivity close below, but well above the 95% confidence level.

In previous studies, localized areas of synchrony were recorded in the seizure onset zone and removal of these regions improved seizure outcome [21], [33]. These were recorded using grids with electrode contacts which were relatively close (approximately 1 cm apart) pointing to the fact that these discrete regions of synchrony in the seizure onset zone are highly localized. In fact, Ortega and collaborators observed that surgical resection of highly localized regions of synchrony detected in electrocorticography predicted better outcomes with epilepsy surgery, but surgical resection of broadly distributed regions of synchronization clusters did not [20]. Similarly, in recordings from intracranial EEG electrodes placed in various temporal lobe structures, we found heterogeneous functional connectivity between widely separated regions in the temporal lobe in patients who had recurrent seizures after surgery. We also saw high synchrony (correlation) in one patient who was seizure free after temporal lobectomy but was falsely predicted to have recurrent seizures. It is possible that in this patient we recorded SEEG signals from one of the hyper-synchronous clusters reported by Ortega et al., due to a wider seizure onset zone or that the electrodes were closer together compared to other patients.

We defined a favorable outcome as complete seizure freedom and any recurrence of seizures were classified as an unfavorable outcome. We did not classify outcome further into more subgroups because of low sample size.

Two patients who continued to have seizures after temporal lobectomy had higher values of connectivity. We speculate that the seizure onset zone in those patients extended outside the temporal lobe and was not removed by a standard anterior temporal lobectomy. It is also possible that these two patients had multifocal epilepsy. Thus our analysis showed increased connectivity in the temporal lobe even though they had a poor surgical outcome.

Previous studies reported increased connectivity in the epileptic brain as analyzed from intracranial EEG signals [34]–[36]. Some of those studies analyzed connectivity *within* the seizure onset zone. They also varied widely in the method of analysis of connectivity, the anatomical proximity of the regions of interest, the control group selected, the number of subjects and the measure used to correlate with seizure outcome. For example, Bettus and colleagues analyzed the nonlinear correlation values in intracranial EEG averaged over six different combinations in 4 different structures (anterior and posterior hippocampus, entorhinal cortex and amygdala) and compared it between patients with TLE and extra temporal epilepsy [34]. Fluctuations in functional connectivity in the early years of epilepsy may be another reason for this apparent contradiction [37].

Recent studies with MEG have reported a focal increase in coherence in the epileptogenic zone in patients with intractable TLE [38]. In contrast, most of the fMRI studies found lower connectivity in the epileptogenic side in TLE [39], [40]. Pittau et al showed that in mesial TLE, the amygdala and the hippocampus showed decreased functional connectivity with wide spread brain regions including the default mode network, ventromesial limbic prefrontal regions, contralateral mesial limbic structures and pons [41]. But focal regions of increased coupling were also noted in fMRI studies on 5 patients with intractable epilepsy [42]. A recent fMRI study using Granger causality, found altered causal relationship between the epileptogenic zone and other cortical networks [43]. The disparity of results obtained with different recording techniques is not surprising considering that EEG, MEG and fMRI detect different physiologic signals with different spatial and temporal scales. Understanding these discrepancies is an important challenge for future studies.

All our patients had a standard procedure with consistent amount and location of tissue removed. This means that even though the seizure onset zone, as concluded from invasive and noninvasive evaluation was limited to one temporal lobe structure like the amygdala or the hippocampus, a standard temporal lobectomy was performed. We tried to analyze the mean connectivity between different structures in the diseased temporal lobe which not only included the seizure onset zone but also a large area around it. This essentially allowed us to calculate the strength of the connections of the seizure onset zone with the surrounding region which may be diseased, but not necessarily generating seizures and also the connectivity *between* these surrounding areas.

The patient population that we selected was complicated enough to undergo invasive evaluation and the results may not be applicable to many patients with mesial temporal sclerosis who undergo surgery without an invasive evaluation. We feel that it is this specific group of patients with complicated temporal epilepsy who needs better measures to predict outcome. We tried to limit the effect of the type of epilepsy on the network dynamics by only selecting patients with TLE. But TLE is heterogeneous and its effect on network connectivity can vary, which we did not account for.

How does our study differ from the previous studies measuring connectivity and surgical outcome in epilepsy? In addition to the strength of connections which has been studied extensively, we applied a measure of variability of connectivity in our calculations which has not been used thus far to predict surgical outcome in epilepsy. We show that adding standard deviation, a simple measure of variability, to the mean of the correlation coefficient improves predictability of outcome. Analysis of loss of neuronal complexity has been used to lateralize unilateral TLE and to predict outcome in neocortical lesional epilepsy [24], [25]. Loss of variability and increased excitability was recently noted in virtual brains modeled to simulate pathological states like epilepsy [23]. The correlation matrices of the patients give a strikingly visual representation of decrease in strength and loss of heterogeneity of connectivity in patients with a good outcome (Figure S2).

In conclusion, our results thus demonstrate the promising potential of a correlation-based measure of functional connectivity from intracranial EEG to predict surgical outcomes after TLE surgery. Prospective studies with a larger patient sample are needed to determine further applicability of this measure as a diagnostic tool.

## Supporting Information

Figure S1 **Coronal sections showing color-coded parcellation units of the temporal lobe. A)** Superior temporal gyrus (red), middle temporal gyrus (green), inferior temporal gyrus (orange), fusiform gyrus (white), parahippocampal gyrus (blue), hippocampus (black). **B)** Temporal pole. **C)** Amygdala. **D)** Hippocampus tail.
(TIF)

Figure S2 **Connectivity matrices for all patients.** Each matrix is the average of connectivity matrices for three different segments of interictal activity. The sign (+) means positive surgical outcome and (−) means negative outcome.
(TIF)

Figure S3 **Averaged connectivity values between areas for positive and negative surgical outcomes.** Positive outcome is generally associated with low connectivity values except for the interaction between the hippocampus and amygdala. High connectivity values are in general associated with a negative outcome, especially between the parahippocampal gyrus and the temporal pole. Black indicates absent pairs in our data.
(TIF)

Table S1 **Clinical characteristics of the patient cohort.** None of the 20 parameters are significantly different between the groups with positive and negative surgical outcome.
(PDF)
