# Supplementary figures and images for: Functional Connectivity Estimated from Intracranial EEG Predicts Surgical Outcome in Intractable Temporal Lobe Epilepsy
Source: PLoS One. 2013 Oct 30;8(10):e77916. doi: 10.1371/journal.pone.0077916 (PMC3813548; doi:10.1371/journal.pone.0077916)

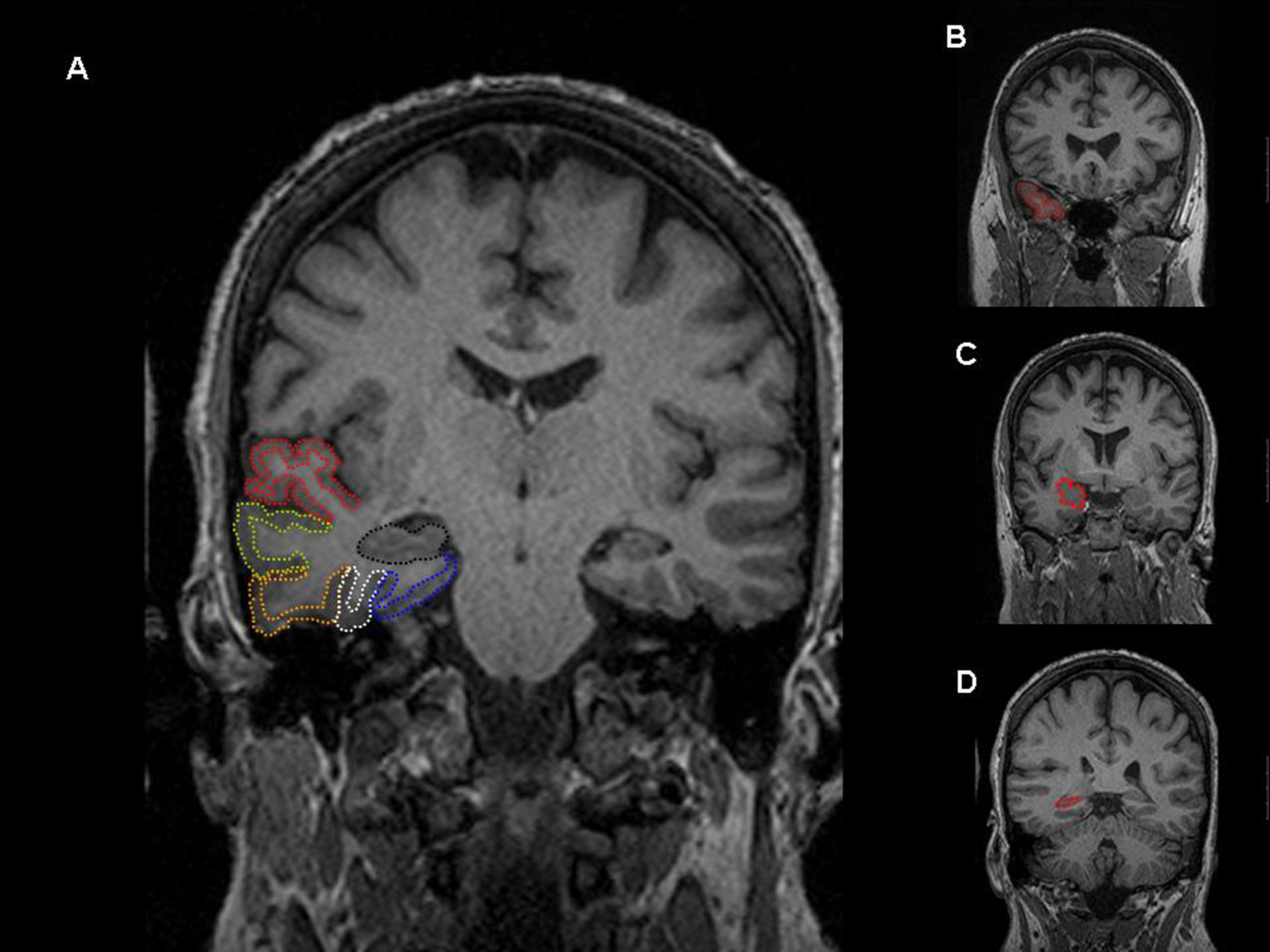

Supplement: Figure S1 — Coronal sections showing color-coded parcellation units of the temporal lobe. A) Superior temporal gyrus (red), middle temporal gyrus (green), inferior temporal gyrus (orange), fusiform gyrus (white), parahippocampal gyrus (blue), hippocampus (black). B) Temporal pole. C) Amygdala. D) Hippocampus tail. (TIF) [file pone.0077916.s001.tif]

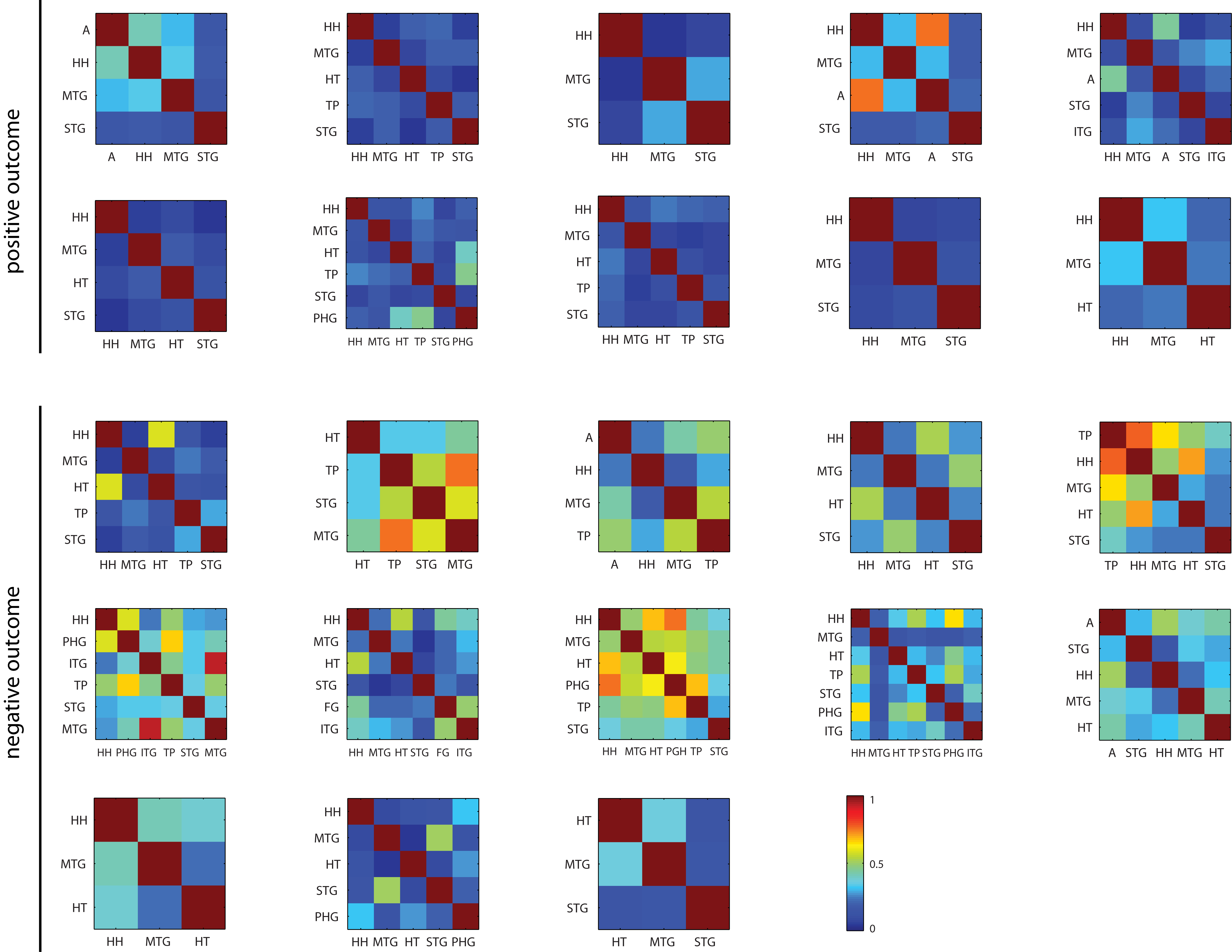

Supplement: Figure S2 — Connectivity matrices for all patients. Each matrix is the average of connectivity matrices for three different segments of interictal activity. The sign (+) means positive surgical outcome and (−) means negative outcome. (TIF) [file pone.0077916.s002.tif]

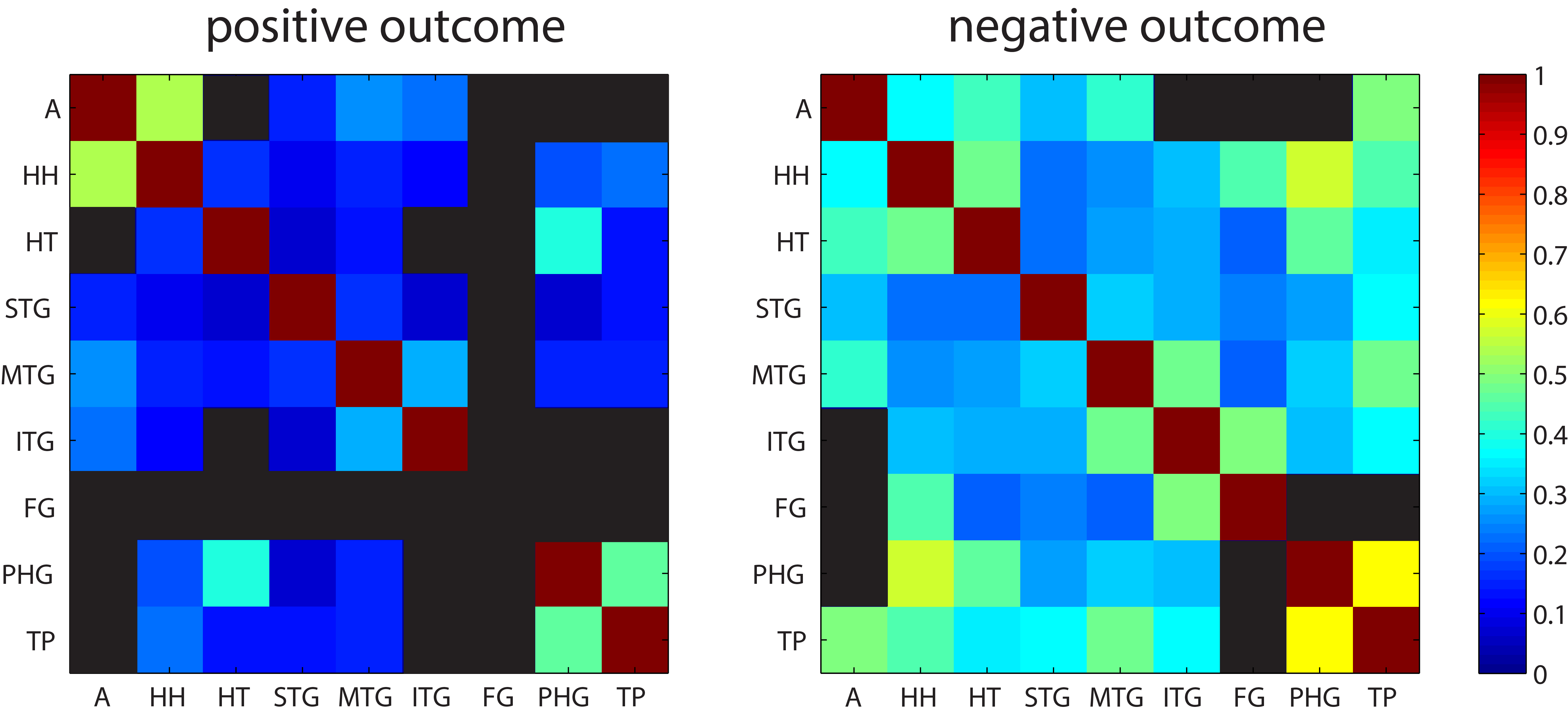

Supplement: Figure S3 — Averaged connectivity values between areas for positive and negative surgical outcomes. Positive outcome is generally associated with low connectivity values except for the interaction between the hippocampus and amygdala. High connectivity values are in general associated with a negative outcome, especially between the parahippocampal gyrus and the temporal pole. Black indicates absent pairs in our data. (TIF) [file pone.0077916.s003.tif]
