# Supplementary material for: Functional Connectivity Estimated from Intracranial EEG Predicts Surgical Outcome in Intractable Temporal Lobe Epilepsy
Source: PLoS One. 2013 Oct 30;8(10):e77916. doi: 10.1371/journal.pone.0077916 (PMC3813548; doi:10.1371/journal.pone.0077916)
Supplement: Table S1 — Clinical characteristics of the patient cohort. None of the 20 parameters are significantly different between the groups with positive and negative surgical outcome. (PDF) [file pone.0077916.s004.pdf]

Supplemental Table: Clinical characteristics of the cohort (n=23). P-value was calculated using Wilcoxon rank-sum test or Fisher's exact t-test with Lancaster's mid P correction if appropriate to compare between the group with seizure freedom after surgery and the group with seizure recurrence after surgery.

Patient characteristics	Overall group, (n=23)	Seizure freedom (n=10)	Seizure recurrence (n=13)	P-value
1. female (%)	12 (52.2)	6 (60)	6 (46.2)	0.54
2. Mean age of onset, years (SD)	16.9 (14.9)	20.5 (18.7)	14.08 (11.3)	0.44
3. Mean age at surgery, years (SD)	34.04 (14.4)	38.9(14.3)	30.3 (13.9)	0.13
4. Mean duration of epilepsy, years (SD)	15.2 (10.8)	18.4 (14.1)	12.7 (7.0)	0.33
5. Two or more risk factors for epilepsy	8	3	5	0.84
6. Previous epilepsy surgery	3	1	2	0.78
7. Multiple seizure types (%)	9 (39.1)	4 (40)	5 (38.5)	0.83
8. Mean pre-operative seizure frequency per month (SD)	6.9 (7.2)	4.2 (3.6)	9.0 (8.7)	0.15
9. Mean number of antiepileptic medications tried (SD)	6.8	6.5	7.1	0.35

**Radiological characteristics**				
10. **MRI brain:** Normal (%)	15 (65.2)	5 (50)	10 (76.9)	0.25
Abnormal: temporal	4	2 (20)	2 (15.4)	
Abnormal: extra temporal	4	3 (30)	1 (7.7)	
11. **PET:** Normal	1	0	1	0.71
Abnormal	22	10	12	
12. **SPECT:** Temporal	4	3	1	0.18
Ipsilateral extratemporal	3	0	3	
Contralateral	5	2	3	
**EEG characteristics**				
**13. Noninvasive EEG:Interictal spikes [2]** None	8	4	4	0.82
Temporal spikes only	11	5	6	
Extra temporal spikes	1	0	1	
Contralateral	4	1	3	
**14. Noninvasive EEG: Ictal rhythm [3]** Temporal	14	6	8	0.42
Lateralized	7	4	3	

**15. Invasive EEG: Ictal onset zone** [3] Mesial temporal only	13	6	7	0.63
Lateral temporal only	4	1	3	
Multifocal	4	1	3	
16. Surgery in the dominant temporal lobe [4]	10	2	8	0.06
**Pathology**				
17. Focal cortical dysplasia	9	5	4	0.46
18. Hippocampal sclerosis	1	0	1	-
19. Dual pathology (Hippocampal sclerosis or remote infact + focal cortical dysplasia)	5	3	2	-
20. Remote infarct	1	0	1	-

Notes

[1] Risk factors for epilepsy: Febrile seizures, Head trauma with loss of consciousness, History of epilepsy in first degree relatives, Cerebral infections, Brain tumor, Stroke, perinatal complications, developmental delay.

[2] Some patients had more than one type of Interictal spikes.

[3] Some patients had more than one type of ictal rhythms

[4] Dominance ascertained by handedness OR Wada test / functional MRI/cortical stimulation when available
